# Development and Validation of a Machine Learning Model for Early Prediction of Sepsis Onset in Hospital Inpatients from All Departments

**DOI:** 10.3390/diagnostics15030302

**Published:** 2025-01-27

**Authors:** Pierre-Elliott Thiboud, Quentin François, Cécile Faure, Gilles Chaufferin, Barthélémy Arribe, Nicolas Ettahar

**Affiliations:** 1 PREVIA MEDICAL, 69007 Lyon, France; pierre-elliott.thiboud@previa-medical.com (P.-E.T.); quentin.francois@previa-medical.com (Q.F.); bart.arribe@previa-medical.com (B.A.); 2 ALLYANE, 69004 Lyon, France; g.chaufferin@allyane.com; 3 Service de Maladies Infectieuses et Tropicales, Centre Hospitalier de Valenciennes, 59300 Valenciennes, France; ettahar-n@ch-valenciennes.fr

**Keywords:** sepsis, machine learning, algorithm, early prediction

## Abstract

**Background:** With 11 million sepsis-related deaths worldwide, the development of tools for early prediction of sepsis onset in hospitalized patients is a global health priority. We developed a machine learning algorithm, capable of detecting the early onset of sepsis in all hospital departments. **Methods:** Predictors of sepsis from 45,127 patients from all departments of Valenciennes Hospital (France) were retrospectively collected for training. The binary classifier SEPSI Score for sepsis prediction was constructed using a gradient boosted trees approach, and assessed on the study dataset of 5270 patient stays, including 121 sepsis cases (2.3%). Finally, the performance of the model and its ability to detect early sepsis onset were evaluated and compared with existing sepsis scoring systems. **Results:** The mean positive predictive value of the SEPSI Score was 0.610 compared to 0.174 for the SOFA (Sepsis-related Organ Failure Assessment) score. The mean area under the precision–recall curve was 0.738 for SEPSI Score versus 0.174 for the most efficient score (SOFA). High sensitivity (0.845) and specificity (0.987) were also reported for SEPSI Score. The model was more accurate than all tested scores, up to 3 h before sepsis onset. Half of sepsis cases were detected by the model at least 48 h before their medically confirmed onset. **Conclusions:** The SEPSI Score model accurately predicted the early onset of sepsis, with performance exceeding existing scoring systems. It could be a valuable predictive tool in all hospital departments, allowing early management of sepsis patients. Its impact on associated morbidity-mortality needs to be further assessed.

## 1. Introduction

Sepsis remains a major cause of morbidity and mortality worldwide, with an estimated 49 million cases of sepsis and 11 million sepsis-related deaths occurred worldwide in 2017, accounting for approximately 20% of all-cause deaths globally [1,2]. Future projections predict that the number of sepsis cases will double in the next 50 years due to an ageing population [1,3]. In the past few decades, the high prevalence of sepsis and its high economic impact have led to the development of several projects intended to allow better recognition and more accurate description of the course of the disease [4]. The new Sepsis-3 definitions underline the concept of a dysregulated immune response resulting in potentially modifiable life-threatening organ dysfunction [5]. In 2017, the World Health Assembly and the World Health Organization made a sepsis global health priority by adopting a resolution to improve, prevent, diagnose, and manage sepsis [6]. Indeed, the development of tools to predict sepsis, and particularly the early detection of sepsis, is critical as a one-hour delay in sepsis diagnosis is associated with a 7% reduction in survival [7]. There are several international scoring systems for sepsis detection including the Systemic Inflammatory Response Syndrome (SIRS) [8], the Sepsis-related Organ Failure Assessment (SOFA) scoring system [9,10], the Modified Early Warning Score (MEWS) [11,12], and quick SOFA score (qSOFA) [5,12]. It has however been stated that no scoring system had both high sensitivity and specificity for predicting the accuracy of mortality in patients with suspected sepsis [13]. Therefore, it has been recommended against using qSOFA compared to SIRS or MEWS as a single screening tool for sepsis [14,15]. Although useful these manual screening tools are found to suffer from limitations that can induce diagnostic and plan of care delays and impact the patient outcomes.

In contrast, automated screening tools that have been recently developed over the last few years have the potential to decrease diagnostic delays and increase screen accuracy. Several predictive models for sepsis have been developed using machine learning (ML) algorithms [16,17,18,19,20,21]. Both retrospective and prospective studies have shown that implementation of the InSight^®^ (Dascena, San Francisco, CA, USA) algorithm [20,22] for sepsis management reduced sepsis-related hospital length of stay by 10% [23] or by 2.3 days [20]. More recently, a 32% reduction in hospital length of stay has been reported in a multi-site prospective real-world data study [22]. However, the detection of sepsis by ML algorithms is still a work in progress as they can miss up to 67% of sepsis cases [24]. Currently, there is limited real-world implementation of these models in clinical settings. To ensure these tools can be reliably used across different patient populations, more clinical implementation studies are needed [17]. Notably, the main limitation of these predictive models is the lack of external validation to ensure reproducibility and generalizability [25], while most of them have focused on intensive care unit (ICU) populations [20].

Retrospective studies have shown that machine learning models can effectively predict sepsis onset with strong accuracy and the models’ success largely depends on incorporating clinically relevant variables for sepsis detection [17]. However, the development and implementation of sepsis prediction models with datasets of hospital inpatients from all departments is still limited. Furthermore, more efficient prediction tools are still warranted to have a significant impact on the survival of sepsis patient. The aim of this study was to develop and validate a ML model, the SEPSI Score algorithm, to accurately predict the early onset of sepsis in hospitalized patients from all departments.

## 2. Materials and Methods

### 2.1. Study Design and Setting

The data were retrospectively collected from the electronic health records (EHR) of hospital inpatients admitted to all departments of the Hospital of Valenciennes (CHV, Valenciennes, France) (including intensive care unit, emergency department, surgical department, and all hospital departments where sepsis cases occurred), for both the training and the study datasets.

#### 2.1.1. Study Population

Patients were selected based on the following inclusion/exclusion (i./e.) criteria: (i.1) Adult patient (18 years old or older); (i.2) At least one SOFA-related Observation recorded; (i.3) At least five out of six vital signs documented; (i.4) Length of stay between 2 and 100 days (inclusive); (i.5) Onset of sepsis detected by a 2-point increase in the SOFA score, at least 3 h after hospital admission; (e.1) Missing International Classification of Diseases 10th edition (ICD-10) data; (e.2) Gender unknown; (e.3) No Observation recorded for vital signs and laboratory results.

#### 2.1.2. Data Source

Data were extracted using a hospital-hosted Fast Healthcare Interoperability Resources (FHIR) server, implemented and connected to the hospital EHR and the ICD-10 coding solution, a standardized system used to code diseases and medical conditions (morbidity) data, including sepsis [26]. In this study, we use the FHIR terminology, where an ‘Encounter’ is a single patient stay from admission to discharge and an ‘Observation’ is a time-related measurement (vital sign, laboratory result, …).

### 2.2. Procedures

The ML model was constructed using a gradient boosted trees approach, trained with the data of a first cohort called the “training dataset”, then validated with a set of data from another cohort of patients called the “study dataset”.

#### 2.2.1. Predictors and Other Variables

Three types of variables were collected for the development of the algorithm: temporal, medical history, and demographic. The selection of these variables was based on the review of the existing literature [16] and determined in collaboration with an expert physician. It was also driven by a combination of relevance, data quality, and availability in the electronic health record (EHR).

For time-related data, 15 predictors were selected:

- Vital signs (heart rate—HR, respiratory rate—RR, diastolic blood pressure—DBP, systolic blood pressure—SBP, oxygen saturation—SpO_2_, and body temperature);
- Laboratory values (creatinine, lactate, white blood cells—WBC, platelets, bilirubin, diuresis, partial pressure of oxygen—PaO_2_, and fraction of inspired oxygen—FiO_2_);
- Assessment of level of consciousness (Glasgow Coma Scale—GCS).

Medical history, consisting of ICD-10 codes to determine existing comorbidities, were also included as variables.

Finally, the patient’s age, gender, and month of hospital admission were added.

#### 2.2.2. Outcome Variables

Sepsis patients were binary classified as non-sepsis and sepsis cases in the datasets. Thus, the outcome variable is the presence of sepsis, and the output of the model is the probability that the selected window ends with the onset of sepsis. While the Sepsis-3 definition represents the most recent consensus on sepsis classification, it presents limitations for early detection purposes. Due to the methodological challenges in precisely determining sepsis onset, particularly in case of preventive antibiotic treatments or microbiology, the gold standard for Encounter classification in the training dataset was the presence of ICD-10 codes corresponding to sepsis in the medical record (Table 1). The decision to prioritize classification certainty over exhaustiveness was particularly crucial during the learning phase of the model development, as the accuracy of the training data directly impacts model performance. The prognosis of sepsis was determined with the SOFA scoring system, which is based on six sub-scores (from 0—normal to 4—most abnormal) assessing the respiratory, neurological, cardiovascular, hepatic, renal, and coagulation systems [9]. The time of “sepsis onset” was defined as a 2-point increase in SOFA score. In the study dataset, all suspected sepsis (flagged by ICD-10 codes) were retrospectively reviewed by a medical expert, who provided a clear decisive opinion, ensuring high homogeneity in the reviews, which is crucial for aligning the model with clinical objectives. The gold standard for labelling sepsis cases was the expert’s judgement, based on a critical review of the patient’s medical record. The physician also gave an exact time of onset of sepsis, later called “expert onset”.

#### 2.2.3. Preprocessing of the Datasets

**Figure 1:**
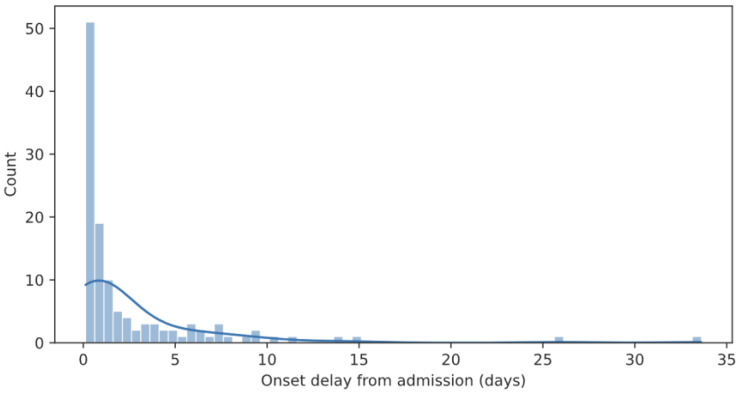
Distribution of time of sepsis onset from admission (expressed in days).

Preprocessing of the extracted raw data was required before the datasets could be fed into the ML algorithm. The preprocessing steps consisted of the following: (i) In the case of multiple Encounters for a patient, each Encounter was considered separately; (ii) Time-stamped data were binned to round hours, using the last available Observation value; (iii) Missing values were imputed using a simple fill-forward strategy (the last known value is propagated until a new value is available); (iv) Temporal predictors were derived from a single 3 h period, spanning 2 h before and up to the onset of sepsis; (v) Metrics were calculated over the 3 h periods (means with standard deviation, median, minimum, and maximum, last value). The 3 h window of the preprocessing step was selected as the best performing set of parameters from a pool of possible values, as described in Section 2.2.6. For non-septic patients, a time point was selected randomly during the hospital stay following a Pareto distribution (Type II, with α = 20 and λ = 1). With this distribution, “non-septic time points” are more often selected in the beginning of the patient stay, simulating the distribution of sepsis onsets, which is displayed in Figure 1.

#### 2.2.4. Training Dataset

**Figure 2:**
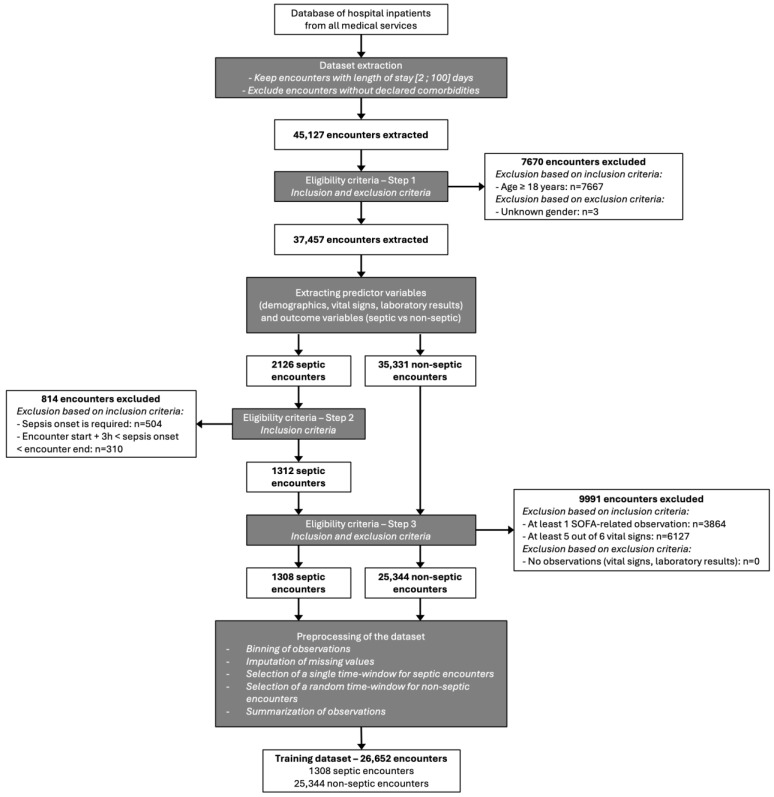
Flow chart of training dataset.

To build the training dataset, we extracted 45,127 Encounters from patients hospitalized between 6 February 2020 and 31 July 2021 (Figure 2). Patients diagnosed with sepsis were identified based on the registration of ICD-10 codes, mentioned in the EHR. The rule used to define the onset of sepsis was the first 2-point increase in the SOFA score as the primary objective of this study was to develop an early detection system for sepsis. The 3 h periods were then set for the temporal predictor variables. Patients were selected based on the eligibility criteria (Figure 2) and a preprocessing procedure was applied to the extracted data. Finally, the training dataset included a total of 26,652 Encounters with a sepsis prevalence of 4.9% (*n* = 1308 sepsis).

#### 2.2.5. Study Dataset

**Figure 3:**
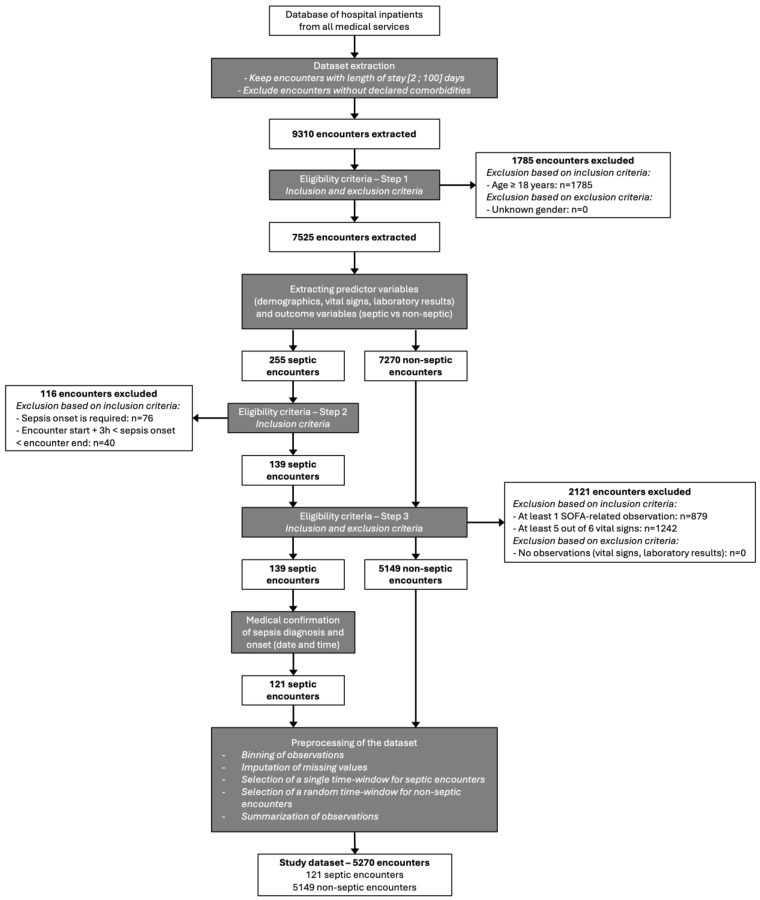
Flow chart of study dataset.

The algorithm was then validated in another retrospective cohort of hospitalized patients. To build the study dataset, we extracted 9310 Encounters from patients hospitalized between 1 August 2021 and 30 November 2021 (Figure 3). After verification of eligibility criteria, the final study dataset included 5270 Encounters of which 121 sepsis patients (2.3%) identified by ICD-10 codes were confirmed by an expert physician. The date and time of the first signs of sepsis were determined after a complete review of the patients’ medical records (i.e., anamnestic parameters, clinical parameters (semiology, vital parameters), biological parameters, time of blood culture collection, and time of blood culture positivity). Among the 121 Encounters of septic patients, 77 sepsis events were reported at admission, and 44 sepsis events were identified during hospitalization.

As with the training dataset, 3 h periods were selected for temporal predictors and the data were then pre-processed to create the final dataset.

#### 2.2.6. Implementation Details on SEPSI Score

The binary classifier SEPSI Score (inpatients classified as having or not having sepsis) was constructed using a gradient boosted trees approach and a simple binary logistic loss function, implemented in Python software (v 3.10.8, [27]) using the XGBoost library (v 1.7.3, [28]). Tree-based models such as XGBoost are still state-of-the-art for medium-sized tabular datasets [29]. The hyperparameters were the default XGBoost parameters except colsample by tree (0.8), with a learning rate of 0.3 and a maximum tree depth of 6. As mentioned in the preprocessing section, a window length of 3 h was used for time-related variables.

All these parameters (for the preprocessing steps, the resampling and ML model) were found after an hyperparameter search on the training dataset. This optimization process allowed to select the best performing set of parameters from a pool of possible values. For example, the 3 h window of the preprocessing step was selected from the set of 3 h, 6 h, and 12 h window sizes.

A stratified shuffle split was performed to extract 10% of the training dataset as the test set and a stratified 5-fold cross-validation was performed on the remaining 90% to estimate the generalization ability of the model. This stratified shuffle split was created such that prevalence was identical in training and test datasets (4.9%).

Predictions of SEPSI Score were generated using all the data described above and, in parallel, an expert physician performed sepsis prediction based on the same parameters. All scores were calculated every hour.

### 2.3. Evaluation of the Model

For comparisons, three time points were considered: in septic Encounters, the time of “sepsis onset” defined as a 2-point increase in SOFA score and the time of “expert onset” defined as the time of medically confirmed onset; in non-septic Encounters, the random “time point”.

#### 2.3.1. Performance Metrics

All performance metrics of SEPSI Score model reported in the manuscript are computed on the defined window (first degradation detected through SOFA score for sepsis patients and random window for non-sepsis). The performance of SEPSI Score was evaluated in terms of specificity, positive predictive value, and sensitivity at the time of sepsis onset, and other metrics as described below.

The ROC (receiver operating characteristic) curve represents the interaction between sensitivity and specificity. AUROC is the area under the receiver operating characteristic curve at the time of sepsis onset. The area under this curve represents the overall accuracy of a test, with a value approaching 1.0 indicating a high sensitivity and specificity. As the dataset was imbalanced (less than 5% of sepsis cases), we also considered as performance metrics the more informative PRC (precision–recall curve) at sepsis onset [30]. AUPR is the area under the precision–recall curve at the time of sepsis onset. The PRC plot shows positive predictive values for corresponding sensitivity values. It is like the AUROC but while the baseline is fixed with ROC, the baseline of PRC is determined by the ratio of positives and negatives.

Other metrics, such as F1 score (harmonic mean of positive predictive value and sensitivity), accuracy, negative predictive value, miss rate (false negative rate), and fall-out (false positive rate) were also calculated to evaluate the model performance.

A single prediction was performed for each patient Encounters but as cross-validation was used during training, we have access to multiple models, one for each fold. Thus, these models were used to compute metrics on the held-out study dataset. We then calculated the mean and standard deviation for each of the metrics mentioned above.

#### 2.3.2. Comparison with Existing Scores

The classification performance of SEPSI Score was then compared with the currently used scoring systems, SOFA score, MEWS, qSOFA, and SIRS criteria, which are commonly used by clinical practitioners to diagnose sepsis and predict mortality due to infection. These scores were calculated at hourly intervals across all patient Encounters in the dataset. The scores were calculated in the same time windows as those generated by the SEPSI Score, and their base value is 0. For comparative analysis with the SEPSI score machine learning models, the time point considered was the last time point of the window for which SEPSI Score generated an alert for the risk of sepsis. Positive predictions of the currently used scoring systems were based on their respective thresholds, i.e., a difference of 2 points for SOFA, a difference of 3 points for MEWS, the presence of 2 criteria for qSOFA and the presence of 2 criteria for SIRS.

#### 2.3.3. Detection of Early Sepsis Onset

The early detection of sepsis risks leading to the improvement of patient clinical outcomes, was evaluated 3 h before sepsis onset. Predictions up to 3 h before sepsis onset were computed for both sepsis and non-sepsis Encounters to determine AUROC and AUPR metrics, as previously described [18]. The predictive capabilities of SEPSI Score were compared with existing scores at the same time point. The targeted value was set at: AUROC_3h before sepsis onset_ = 0.70 ± 0.15, based on the state of the art.

The AUPR metric was also considered up to 3 h before sepsis onset [30].

The time of sepsis onset estimated by the SEPSI Score was further analyzed excluding sepsis present on admission to focus only on sepsis diagnosed during hospitalization (*n* = 44). The delay between the sepsis onset (defined as the time of the first degradation detected through SOFA score for sepsis patients) and the expert onset (the time of sepsis onset which was validated by the physician, based on the review of the patients’ medical records and its own experience) was then considered. The number of patients Encounters who were detected for sepsis at least 48 h before their medically confirmed onset (expert onset) was then determined, among these 44 Encounters.

#### 2.3.4. Statistical Analysis

We aimed to demonstrate a minimum effect size of 0.05 with an alpha risk of 0.05 and a beta risk of 0.05, using a *t*-test for comparison of means. Based on these parameters, a minimum sample size of 4331 was calculated.

All statistical analyses were performed using Python software with the pandas (v 1.5.3, [31]) and scipy (v 1.12.0, [32]) libraries. Continuous data were described as mean (SD) or median (Q1–Q3 or min–max) and categorical data were presented as frequencies (percentages). *p*-values were calculated using a binomial test adjusted by the Benjamini–Hochberg method.

### 2.4. SEPSI Score Among Other Advanced ML Models

Recent advances in machine learning have yielded several commercial sepsis prediction systems, each employing distinct methodologies and demonstrating varying levels of performance. Among these, notable solutions include InSight^®^ by Dascena (San Francisco, CA, USA) a machine learning system for sepsis prediction, first evaluated on the MIMIC-III ICU dataset, which demonstrated good performance metrics (AUROC: 0.880, APR: 0.595 Sensitivity: 0.80, Specificity: 0.80) [21]; Sepsis ImmunoScore™ (Prenosis, Chicago, IL, USA), employing a 22-parameter analysis system (AUROC: 0.83, AUPR: 0.61) [33]; NAVOY^®^ (AlgoDx AB, Stockholm, Sweden) an ICU-focused system showing reliable performance (sensitivity: 0.80, specificity: 0.78) [19]; and VFusion™ Sepsis by Vivace Health Solutions (Cardiff, CA, USA), which achieves impressive accuracy metrics (AUC: 0.91, sensitivity: 0.814, specificity: 0.88) [34]. Direct performance comparisons between existing machine learning systems for sepsis prediction should be interpreted with caution due to their different clinical contexts and validation settings [17]. Therefore, the performance metrics of the SEPSI Score will only be discussed with respect to those of the other ML solutions described in the scientific literature, as opposed to the comparative analysis with currently used scoring systems such as SOFA, qSOFA, MEWS, and SIRS.

## 3. Results

### 3.1. Study Cohort Characteristics

The study dataset consisted of 5270 Encounters, with a total of 121 cases of sepsis. Aggregated patient demographics at inclusion were a mean age of 65.7 (17.4) years and 50.2% of male (Table 2).

**Figure 4:**
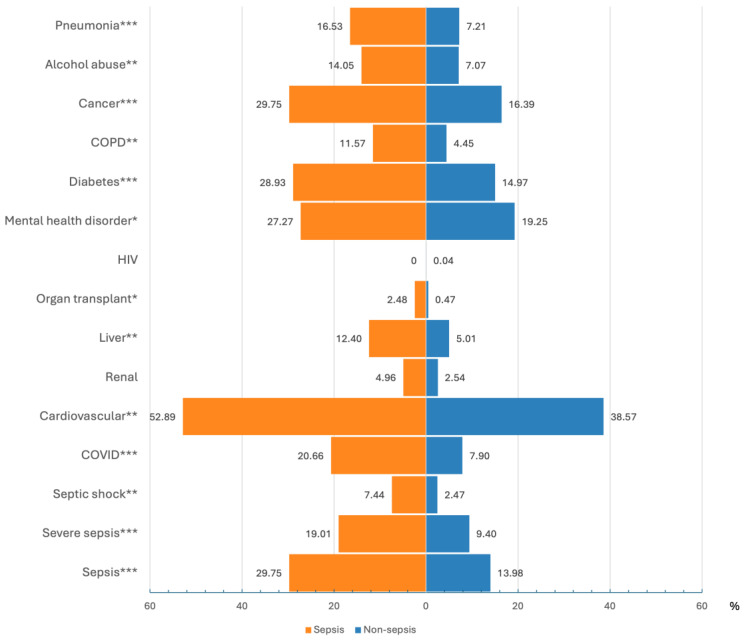
Comorbidities repartition between populations (sepsis versus non-sepsis). Significance is shown as * in the comorbidity group name, with the following meaning: ***: *p*-value < 0.001; **: *p*-value < 0.01; *: *p*-value < 0.05; COVID-19, coronavirus disease; HIV, human immunodeficiency virus; COPD, chronic obstructive pulmonary disease.

The most common comorbidities in the study cohort were cardiovascular disease (38.9%), mental health disorder (19.4%), cancer (16.7%), diabetes (15.3%), and previously diagnosed sepsis (14.3%). In the sepsis subpopulation, the top five comorbidities were identical (although in a different order), but with a higher prevalence than in non-sepsis patients (Figure 4). The differences in the prevalence of comorbidities were all statistically significant at the 0.05 level, except for HIV and renal disease.

Some vital signs were obtained immediately after admission, with a mean delay of 3 h for HR, temperature, and BP. Vital signs were recorded frequently (on average every 5 to 56 h), but laboratory results were not regularly available, with delays between measurements of days (creatinine, white blood cells, platelets, bilirubin, and diuresis) or weeks (lactate, PaO_2_, and FiO_2_) (Table 2). The mean length of hospital stay was higher for sepsis reported during hospitalization (16.7 days versus 5.9 days, Table 2) and the time of sepsis onset appeared to be within the first 3 days after admission for 75% of sepsis Encounters.

### 3.2. Performance Metrics of the Model

The performance metrics of the model developed with the training dataset demonstrated its ability to correctly classify sepsis-positive cases (positive predictive value: 0.752 [0.015], sensitivity: 0.855 [0.022], specificity: 0.985 [0.001], AUROC: 0.993 [0.001], AUPR 0.873 [0.015]).

The SEPSI Score algorithm was then validated in the study dataset and showed both a high sensitivity of 0.845 [0.018], and a high specificity of 0.987 [0.001] for the detection of sepsis (Table 3). Thanks to the multiple models trained during cross-validation, we were able to provide a mean ± SD for SEPSI Score on the held-out study dataset, opposite of SOFA, qSOFA, SIRS, and MEWS scores for which the computation was unique. SEPSI Score was reliable in correctly classifying sepsis with a mean positive predictive value of 0.610 [0.018], exceeding the performance of competing scores SOFA, qSOFA, SIRS, and MEWS (0.174, 0.175, 0.070, and 0.108, respectively, Table 3).

**Figure 5:**
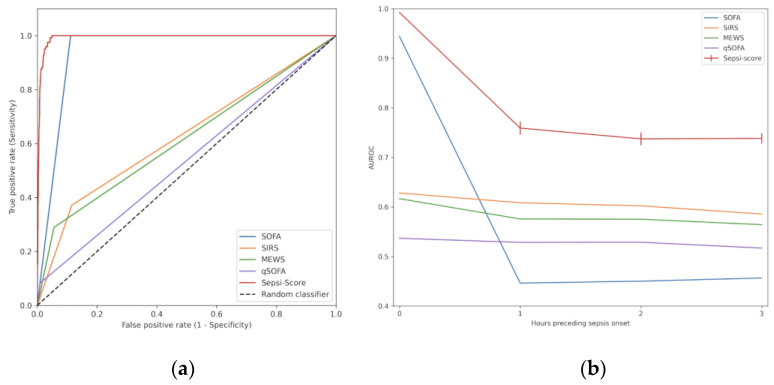
ROC-related metrics for ML models and existing scores: (**a**) ROC curves at sepsis onset for ML model, existing scores, and random model; (**b**) AUROC metrics up to 3 h before sepsis onset for ML model and existing scores.

The juxtaposition of the ROC curves plotting the true positive rates against the false positive rates of the SEPSI Score algorithm and of the aforementioned scores, demonstrates the advantage of our model (Figure 5a).

**Figure 6:**
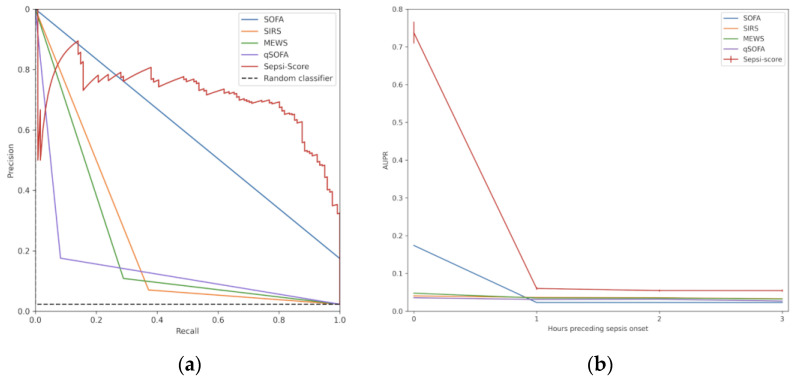
Precision–recall-related metrics for ML model and existing scores: (**a**) PRC at sepsis onset for ML model, existing scores, and random model; (**b**) AUPR metrics up to 3 h before sepsis onset for ML model and existing scores.

Considering the PRC curves (Figure 6a), the positive predictive value of qSOFA, MEWS and SIRS decreased dramatically with increasing sensitivity, whereas the high positive predictive value of SEPSI Score remained constant for sensitivity values from 0.2 to 0.8. At the onset of sepsis, the AUPR value (with a baseline of 0.023) was 0.738 for SEPSI Score versus 0.174 for SOFA, 0.036 for qSOFA, 0.041 for SIRS, and 0.048 for MEWS (Figure 6b).

### 3.3. Detection of Early Sepsis Onset

Furthermore, up to 3 h before the onset of sepsis, SEPSI Score was consistently more accurate than any of the scores tested, as shown by the AUROC curves (Figure 5b). These results show that the predictive capabilities of SEPSI Score, AUROC_3h before sepsis onset_ = 0.74, were higher that the predictive capabilities of the SOFA (AUROC_3h before sepsis onset_ = 0.46), the SIRS (AUROC_3h before sepsis onset_ = 0.59), the MEWS (AUROC_3h before sepsis onset_ = 0.56) and the qSOFA (AUROC_3h before sepsis onset_ = 0.52). Moreover, the predictive capabilities were also higher than the limits identified from the state of the art (AUROC_3h before sepsis onset_ = 0.70 ± 0.15). Thus, up to 3 h before sepsis onset, sepsis detection by SEPSI Score was constantly more accurate than all tested scores.

When focusing only on subpopulation of sepsis diagnosed during hospitalization (*n* = 44), close to 75% (*n* = 32) of sepsis onsets were detected by the SEPSI Score algorithm earlier than estimated by the expert physician. Moreover, half (*n* = 21) of sepsis cases were detected by SEPSI Score at least 48 h before their medically confirmed onset (expert onset).

## 4. Discussion

The performance metrics of SEPSI Score are considerable and demonstrate the ability of the model to confidently detect early sepsis onset. The positive predictive value, sensitivity and specificity achieved by the model show not only the correct detection of a large majority of sepsis cases, but also the low number of false positives. Indeed, sensitivity is particularly important in sepsis detection because missing sepsis cases can lead to delays in treatment, resulting in worse patient outcomes. The very high specificity of SEPSI Score involves a lower risk of unnecessary interventions, such as administering antibiotics or triggering the sepsis protocol for non-septic patients. Reducing false positives is crucial in real-world settings to avoid the risk of alarm fatigue and its potential consequences such as over-treatment and resource wastage [21,35].

Moreover, SEPSI Score showed a better performance than the scores commonly used in standard clinical practice to assess sepsis, such as SOFA, qSOFA, SIRS, and MEWS scores. When comparing the SEPSI Score model with existing Early Warning Scores, the specificity shows its limits in this unbalanced setting. Indeed, while these commonly used scores achieved a high specificity, their positive predictive value metrics were comparatively low. This highlights the difference in interpretation between specificity and positive predictive value in the context of an unbalanced dataset [30]. This low positive predictive value also means that eight to nine out of ten alerts can be expected to be false if used for sepsis detection in everyday hospital practice. Therefore, we advocate the inclusion of the positive predictive value metric, or minority class-focused metrics such as AUPR, in sepsis prediction model performance tables.

Other machine learning algorithms have been developed these last few years for predicting sepsis in hospitals, mainly in ICU, and have also shown better predictive abilities compared to current conventional scoring system [16,18,36,37,38,39]. Indeed, InSight^®^ and Navoy^®^ models were both able to detect sepsis in ICU patients with sensitivities of 0.80 and 0.80, and specificities of 0.80 and 0.78 respectively [19,21]. SEPSI Score sensitivity and specificity (0.845 and 0.987 respectively) can thus be considered as very respectable performance metrics when compared to those of the previously cited models. In the same way, Persson et al. in their recent prospective study reported lower positive predictive value and accuracy than those of SEPSI Score [19]. The AUROC and the AUPR measured for the SEPSI Score also showed more interesting values when compared with the same metrics reported for another model, the Sepsis ImmunoScore^TM^, in a prospective observational cohort study [33]. SEPSI Score thus showed better performance metrics than the above cited existing models although they are not fully comparable. Indeed, most of them have been evaluated only in prospective ICU settings [40], whereas SEPSI Score has shown its performance in a wide range of departments from a single hospital, in a retrospective setting. The other ML models have then a limited application to ICU patients, leading to a poor generalizability of the models and limiting their implementation in clinical practice [39]. SEPSI Score is the first model to our knowledge that has been built using data from hospital inpatients from all medical services and is therefore promising to have a better generalizability in clinical practice than the existing ones.

Another important point is that sepsis detection by SEPSI Score was more accurate (AUROC_3h before sepsis onset_ = 0.74) than all tested scores up to 3 h before sepsis onset. A recent study has shown that Navoy^®^ algorithm was also able to predict sepsis 3 h before onset with a high performance (AUROC of 0.80) [19]. However, this latter prediction was limited to ICU patients. Moreover, the ability of SEPSI Score to predict for some patients, the onset of sepsis up to 48 h in advance must be highlighted, as this is a critical window for initiating timely interventions to prevent severe sepsis and septic shock. VFusion^TM^ Sepsis also seems to be able to detect sepsis onset 24 to 48 h before usual care baselines. However, their application is limited to ICU patients, and to our knowledge their results are stated by their website only and not published yet [34]. The other predictive models described in the scientific literature usually focus on detecting sepsis onset 3 to 6 h before clinical recognition, which is still valuable but does not offer the same extended predictive window [19]. The 48 h-early prediction of sepsis obtained with SEPSI Score may provide a more proactive approach, offering clinicians a larger window for intervention and improving patient outcomes.

Resource requirements and potential barriers such as the integration with existing EHR systems and clinical training to implementing the SEPSI Score in real-world clinical settings were analyzed. The initial extraction of data was done in the international standards HL7 FHIR, and therefore facilitates generalization and integration of the SEPSI Score with existing EHR systems. Moreover, the SEPSI Score tool has been developed with a team of clinicians so that it can be used very easily, without extensive training.

Although this study shows promising results for the prediction of sepsis in the hospital, some limitations remain. Indeed, the training and validation settings are favorable to the ML model by selecting time windows for sepsis Encounters at the onset of sepsis, whereas random time windows are selected for non-sepsis Encounters, following a Pareto distribution. To date, many models have been validated on open access databases in the United States [17]. By developing SEPSI Score on datasets like French real-world medical data, we believed that its predictive capabilities would be reproducible and generalizable to other hospital databases. In the present study, the approach aimed at assessing the performance of SEPSI Score before targeting on demonstration of effects on clinical outcomes. This is why this first study on the model only took place in a single hospital center, which provided both the training and validation datasets. This current retrospective design limits our understanding of real-world effectiveness, which would be better evaluated through a prospective study. Indeed, now that the high sensitivity and specificity of the model have been retrospectively demonstrated, a prospective study with integration of SEPSI Score in the daily routine, including several hospital centers, should be carried out to assess the improvement of treatments and outcomes of patients with sepsis, such as those demonstrated with InSight^®^ algorithm [20,22].

## 5. Conclusions

The ability of the algorithm to accurately detect sepsis and predict its early onset has been validated for the first time in a retrospective setting in all departments of a French hospital, with performance metrics exceeding those of currently used tools. Although a further study of the model in a prospective multicenter setting is needed to confirm the model’s robustness and generalizability and its positive clinical outcomes, SEPSI Score seems to be very promising in reducing sepsis-related morbidity and mortality.

## Figures and Tables

**Table 1:** Sepsis coding.

Sepsis Class	Corresponding ICD-10 Codes
Septic shock	R572
Severe sepsis	R651
Sepsis	A021, A227, A267, A327, A427, B377, O85, A400, A401, A402, A403, A408, A409, A410, A411, A412, A413, A414, A415, A418, A419, P3600, P3610, P3620, P3630, P3640, P3650, P3680, P3690

Note: ICD-10 code corresponding to severe sepsis should not be employed anymore (since 2021) and Encounters where sepsis was coded with an outdated ICD-10 code (R651) were excluded from the dataset.

**Table 2:** Study cohort characteristics.

		Sepsis Encounters (*N* = 121)	Non-Sepsis Encounters (*N* = 5149)
Demographics			
Age	Mean ± SD	69.8 ± 12.7	65.6 ± 17.5
Male	*N* (%)	72 (59.5)	2575 (50.0)
Female	*N* (%)	49 (40.5)	2574 (50.0)
Vital signs			
HR (beats per minute)	Mean ± SD	85.6 ± 19.7	81.0 ± 17.6
Hours between measurements	Mean	5	7
Body temperature (°Celsius)	Mean ± SD	37.1 ± 0.8	36.9 ± 0.7
Hours between measurements	Mean	6	8
RR (breaths per minute)	Mean ± SD	21.7 ± 5.7	21.2 ± 6.1
Hours between measurements	Mean	13	56
DBP (mmHg)	Mean ± SD	67.2 ± 14.9	70.9 ± 15.1
Hours between measurements	Mean	5	7
SBP (mmHg)	Mean ± SD	126.1 ± 22.1	128.6 ± 22.2
Hours between measurements	Mean	5	7
SpO_2_ (%)	Mean ± SD	96.5 ± 2.5	96.6 ± 2.6
Hours between measurements	Mean	6	9
Level of consciousness			
GCS score	Mean ± SD	10.1 ± 5.2	11.4 ± 5.2
Hours between measurements	Mean	16	96
Laboratory results			
Creatinine (mg/L)	Mean ± SD	18.3 ± 15.6	12.3 ± 12.0
Hours between measurements	Mean	37	48
Lactate (mg/L)	Mean ± SD	213.6 ± 255.5	180.0 ± 172.4
Hours between measurements	Mean	1655	3134
WBC (mg/dL)	Mean ± SD	11.6 ± 9.7	9.8 ± 7.6
Hours between measurements	Mean	40	54
Platelet count (×10^9^/L)	Mean ± SD	218.2 ± 146.7	258.7 ± 119.2
Hours between measurements	Mean	40	54
Bilirubin (μmol/L)	Mean ± SD	22.2 ± 36.7	11.5 ± 26.5
Hours between measurements	Mean	72	105
Diuresis (mL)	Mean ± SD	409.3 ± 670.3	466.7 ± 563.5
Hours between measurements	Mean	9	22
PaO_2_ (mmHg)	Mean ± SD	90.5 ± 34.9	86.7 ± 39.0
Hours between measurements	Mean	54	247
FiO_2_ (%)	Mean ± SD	42.5 ± 21.0	45.9 ± 22.6
Hours between measurements	Mean	69	525
Length of hospital stay			
Day(s)	Median	16.7	5.9
	Q1; Q3	10.1; 24.9	3.7; 9.5

**Table 3:** Performance metrics of SEPSI Score versus commonly used scores to classify sepsis. Bold indicates the best value per metric.

		Study Dataset *N* = 5270 121 Sepsis/5149 Non-Sepsis				
		SEPSI Score	SOFA	qSOFA	SIRS	MEWS
**AUROC**	Mean ± SD	**0.992** ± 0.001	0.944	0.537	0.628	0.617
	Median	0.992	-	-	-	-
	Min; Max	0.992; 0.993	-	-	-	-
**AUPR**	Mean ± SD	**0.738** ± 0.029	0.174	0.036	0.041	0.048
	Median	0.728	-	-	-	-
	Min; Max	0.711; 0.775	-	-	-	-
**Positive predictive value**	Mean ± SD	**0.610** ± 0.018	0.174	0.175	0.070	0.108
	Median	0.606	-	-	-	-
	Min; Max	0.593; 0.640	-	-	-	-
**Sensitivity**	Mean ± SD	0.845 ± 0.018	**1.000**	0.083	0.372	0.289
	Median	0.851	-	-	-	-
	Min; Max	0.826; 0.868	-	-	-	-
**Specificity**	Mean ± SD	0.987 ± 0.001	0.889	**0.991**	0.884	0.944
	Median	0.987	-	-	-	-
	Min; Max	0.986; 0.989	-	-	-	-
**F1 score**	Mean ± SD	**0.708** ± 0.013	0.297	0.112	0.118	0.158
	Median	0.705	-	-	-	-
	Min; Max	0.699; 0.730	-	-	-	-
**Accuracy**	Mean ± SD	**0.984** ± 0.001	0.891	0.970	0.872	0.929
	Median	0.984	-	-	-	-
	Min; Max	0.983; 0.986	-	-	-	-
**Negative predictive value**	Mean ± SD	0.996 ± 0.000	**1.000**	0.979	0.984	0.983
	Median	0.996	-	-	-	-
	Min; Max	0.996; 0.997	-	-	-	-
**Miss rate** (false negative rate)	Mean ± SD	0.155 ± 0.018	**0.000**	0.917	0.628	0.711
	Median	0.149	-	-	-	-
	Min; Max	0.132; 0.174	-	-	-	-
**Fall-out** (false positive rate)	Mean ± SD	0.013 ± 0.001	0.111	**0.009**	0.116	0.056
	Median	0.013	-	-	-	-
	Min; Max	0.011; 0.014	-	-	-	-

## Data Availability

Restrictions apply to the availability of these data. Data were obtained from the Centre Hospitalier de Valenciennes (France) and are available from the Centre Hospitalier de Valenciennes (France) with the permission of the Centre Hospitalier de Valenciennes (France).
